# Pleuroparenchymal Fibroelastosis in Connective Tissue Disease-Related Interstitial Lung Disease

**DOI:** 10.3390/jcm15082886

**Published:** 2026-04-10

**Authors:** George E. Dimeas, Ilias E. Dimeas, Cathal Doherty, Eamonn Molloy, Zoe Daniil, Cormac McCarthy

**Affiliations:** 1 Department of Internal Medicine, General Hospital of Karditsa, 43100 Karditsa, Greece; 2 Department of Rheumatology, St. Vincent’s University Hospital, D04 T6F4 Dublin, Ireland; cathaldoc360@gmail.com (C.D.); emolloy@svhg.ie (E.M.); 3 School of Medicine, University College Dublin, D04 C1P1 Dublin, Ireland; cormac.mccarthy@ucd.ie; 4 Department of Respiratory Medicine, St. Vincent’s University Hospital, D04 T6F4 Dublin, Ireland; 5 Department of Respiratory Medicine, Faculty of Medicine, University of Thessaly, Biopolis, 41500 Larissa, Greece; zdaniil@uth.gr

**Keywords:** pleuroparenchymal fibroelastosis, connective tissue disease, interstitial lung disease, systemic sclerosis, rheumatoid arthritis, upper-lobe fibrosis, fibroelastosis, high-resolution computed tomography, pneumothorax, endothelial dysfunction

## Abstract

**Background**: Pleuroparenchymal fibroelastosis (PPFE) is a rare fibroelastotic lung disease characterized histologically by dense pleural and subpleural fibrosis with upper-lobe predominance. In clinical practice, diagnosis often relies on characteristic radiologic findings, as surgical lung biopsy is rarely feasible. Unlike idiopathic pulmonary fibrosis, robust radiologic criteria validated against biopsy-proven cohorts remain limited, and the diagnostic performance of imaging alone is incompletely defined. Although initially described as idiopathic, PPFE is increasingly recognized in secondary settings, including connective tissue disease-associated interstitial lung disease (CTD-ILD), where it frequently overlaps with more common fibrotic patterns. **Methods**: We conducted a focused narrative review of the literature on PPFE in CTD-ILD, synthesizing evidence on morphology, epidemiology, clinical course, prognostic implications, and proposed pathobiological mechanisms, with emphasis on distinguishing true PPFE from PPFE-like lesions. **Results**: CTD-associated PPFE is associated with accelerated lung function decline, increased risk of pneumothorax, and poorer outcomes, particularly in systemic sclerosis and rheumatoid arthritis. However, distinguishing true PPFE from radiologic mimics remains challenging, and diagnostic approaches rely heavily on imaging without robust histopathologic validation. Proposed mechanisms include epithelial injury, immune dysregulation, and vascular or lymphatic abnormalities, although causal links remain unproven. Significant gaps persist regarding natural history and therapeutic responsiveness. **Conclusions**: Earlier identification of PPFE in CTD-ILD is important, as misclassification may delay risk stratification and management. Longitudinal imaging, multidisciplinary evaluation, and standardized diagnostic criteria are needed to improve clinical care and guide future research.

## 1. Introduction

Connective tissue disease-associated interstitial lung disease (CTD-ILD) most commonly exhibits lower-lobe-predominant patterns such as nonspecific interstitial pneumonia (NSIP) and usual interstitial pneumonia (UIP) [1]. However, a smaller subset of patients develops a distinct upper-lobe fibroelastotic process that has gained increasing recognition with the broader adoption of high-resolution computed tomography (HRCT) [1,2]. Pleuroparenchymal fibroelastosis (PPFE), although rare, represents a recognizably distinct fibroelastotic phenotype defined by dense thickening of the visceral pleura and subpleural elastotic fibrosis predominantly in the upper lobes. Definitive diagnosis requires histopathologic confirmation; however, surgical lung biopsy is rarely feasible in clinical practice due to procedural risk and patient frailty [1,2,3]. Initially viewed as an idiopathic disorder, PPFE is now understood to occur across a spectrum of secondary settings, including chronic inflammatory diseases, autoimmune conditions, and post-transplant complications [2,3,4].

Within CTD-ILD, PPFE likely remains underrecognized. Radiologic PPFE-like lesions have been reported in up to 19% of CTD-ILD patients, particularly those with systemic sclerosis and primary Sjögren’s syndrome [5]. Histology-based studies reinforce this association: a PPFE pattern of fibrosis is found in a substantial proportion of autoimmune disease-related interstitial lung diseases (ILDs), raising the question of whether chronic immune-mediated injury may predispose to pleuroparenchymal elastotic remodeling [6]. Case reports and series in rheumatoid arthritis (RA), systemic sclerosis (SSc), mixed connective tissue disease, idiopathic inflammatory myopathies, and antineutrophil cytoplasmic antibodies (ANCA)-associated vasculitis similarly describe PPFE as either coexisting with or overshadowing other interstitial patterns [7,8,9]. However, these estimates are derived largely from small, retrospective cohorts, often from tertiary referral centers, and are therefore subject to selection and referral bias, limiting their generalizability.

A critical challenge in interpreting this literature is distinguishing true PPFE from radiologic mimics [5]. Upper-lobe subpleural consolidation, architectural distortion, and volume loss may resemble PPFE but may also reflect benign apical cap, chronic post-infectious scarring, or upper-lobe-predominant UIP traction changes [5]. Diagnosis relies not only on morphology but also on documented progression over time [10,11]. Unlike idiopathic pulmonary fibrosis, robust radiologic criteria validated against biopsy-proven cohorts remain lacking [10], limiting confidence in imaging-based diagnosis. Importantly, several connective tissue disease (CTD) cohorts demonstrate progressive worsening of PPFE-like abnormalities, often accompanied by functional decline and increased rates of pneumothorax, supporting the notion that these lesions represent more than incidental apical findings [5].

Growing evidence suggests that PPFE in CTD-ILD portends worse outcomes. Given its association with accelerated lung function decline, increased risk of pneumothorax, and poorer outcomes, improving diagnostic precision is clinically imperative to enable earlier risk stratification and closer surveillance [12,13]. Nevertheless, uncertainty persists because of heterogeneous definitions, retrospective study designs, and inconsistent radiologic-histologic correlations. Proposed mechanisms include epithelial injury, immune dysregulation, and vascular or lymphatic abnormalities, although these remain incompletely understood [4,14,15,16,17].

This narrative review synthesizes current evidence on PPFE in the context of CTD-ILD, focusing on morphological features, epidemiology across connective tissue diseases, clinical course, prognostic implications, and emerging mechanistic insights to provide clinicians with a structured framework for improved recognition, diagnostic refinement, risk stratification, and multidisciplinary evaluation.

## 2. Materials and Methods

We performed a focused literature review on PPFE in CTD-ILD. We searched PubMed, EMBASE, and Web of Science from inception to December 2025 using database-specific terms for PPFE (including “pleuroparenchymal fibroelastosis”, “PPFE”, and related upper-lobe fibroelastotic terms) combined with CTD/autoimmune/rheumatic disease terms. Titles/abstracts were screened, full texts were reviewed for eligibility, and reference lists of included papers were hand-searched for additional studies. We included peer-reviewed studies and relevant case series/reports given the rarity of PPFE, and we recorded how each paper defined PPFE to keep “PPFE” separate from “PPFE-like” apical lesions where criteria were incomplete. Studies were excluded if they did not provide relevant data on PPFE/PPFE-like lesions in CTD-ILD or focused on unrelated non-pulmonary manifestations. The search yielded 300 records across databases; after removal of duplicates, records were screened for relevance; 230 articles underwent full-text review and 52 studies were included in the final qualitative synthesis. The review was conducted in accordance with the Scale for the Assessment of Narrative Review Articles (SANRA) to ensure methodological rigor and transparency.

## 3. Defining PPFE: A Distinct Morphological Entity

From a historical perspective, the recognition of PPFE as a distinct entity has evolved over time. PPFE was first described by Amitani et al. in 1992 [18] and later defined as a distinct clinicopathological entity by Frankel et al [19]. It was formally recognized in the 2013 ATS/ERS classification of idiopathic interstitial pneumonias. Accordingly, its recognition is based on the integration of characteristic histopathological and radiologic features [4,20].

In terms of histological characteristics, PPFE is characterized by “definite” diagnostic criteria. In routine practice, histopathologic confirmation is uncommon and often not feasible due to procedural risk, and PPFE is most often diagnosed on the basis of characteristic HRCT findings integrated with clinical context [2,4,9]. One of the principal criteria is upper-zone collagenous fibrosis of the visceral pleura. Additional defining features include homogenous subpleural intra-alveolar fibrosis with elastic fiber deposition along the alveolar walls intervening with collagen deposition [7]. Podoplanin-positive myofibroblasts and small-vessel intimal changes have also been reported [2,3,4,6]. The transition to normal lung parenchyma is typically steep and no lesions are present away from the pleura [3]. Intralobular or centrilobular nodules of fibroelastosis and small numbers of fibroblastic foci at the edge of the lesion are indicative of disease progression. Inflammatory changes are disseminated throughout the parenchyma, consisting mostly of lymphocytic aggregates. Aside from the “definite” criteria, findings considered “consistent with” PPFE include the same pattern of intra-alveolar fibrosis as previously described but without the association with pleural fibrosis, not located beneath the pleura or identified in an upper-lobe biopsy specimen [2,3,4,6]. These categories reflect proposed diagnostic criteria used in the literature [2,4,6]. Throughout this review, epidemiological and prognostic findings are interpreted in light of this definitional heterogeneity.

On chest radiographs, marked bilateral apical subpleural opacities are a characteristic feature of PPFE, often accompanied by thickening of the apical portions [5]. With disease progression, the appearance of more bilateral reticular and nodular opacities may lead to an upward displacement of hilar structures. On a lateral chest radiograph, a reduction in the anterior-posterior diameter can be observed [21]. However, chest radiography alone is insufficient for diagnosis, and HRCT is required for accurate characterization. On HRCT, PPFE is characterized by dense subpleural fibrosis in addition to pleural thickening, predominantly concentrated in the upper lobes [5]. Involvement of the lower lobes is milder or absent. These findings are considered “definite” imaging criteria for PPFE. Imaging criteria “consistent with” PPFE include upper-lobe pleural thickening with subpleural fibrosis in cases where lesion distribution is not limited in upper lobes or where features related to a coexisting disease are present elsewhere. Traction bronchiectasis and architectural distortion are frequently observed in addition to the subpleural consolidation, often with an associated reticular pattern [2,9,10].

However, the characteristic radiological findings are not exclusive to PPFE. The apical cap is a radiologic term describing a density in the upper lobe of the lungs accompanied by thickening of the visceral pleura. This fibroelastic scar may be confused in patients with underlying conditions such as tuberculosis, post-radiation fibrosis and lung neoplasms that mimic this lesion. PPFE-related lesions tend to be broader and denser than an apical cap although this is not absolute in every case [5]. Both may be associated with chronic infection and persistent inflammation may, in turn, lead to the development of PPFE-like lesions. A key distinguishing feature is the often non-progressive nature of an apical cap, in contrast to PPFE, in which the pleural thickening is variable in extent, asymmetric and may extend posteriorly into the lung parenchyma [22,23]. For clarity, throughout this review we use the term “PPFE” with caution, distinguishing where possible between histologically confirmed PPFE, radiological PPFE, and PPFE-like lesions, as definitions vary substantially across studies.

## 4. Distinguishing PPFE from Mimics in CTD-ILD

For clinicians, differentiating PPFE from its radiological mimics remains clinically important. Conditions such as asbestosis, fibrosing sarcoidosis, radiation-induced lung disease, and post-infectious fibroelastotic remodeling may present with similar radiological features. Careful clinical correlation and, where available, histological evaluation assist in establishing the correct diagnosis. A negative history of occupational asbestos exposure and the absence of sarcoid-type granulomas on lung biopsy help exclude these alternative diagnoses [4]. In this context, multidisciplinary discussion integrating clinical, radiological, and, where available, histopathological data is essential.

In its early stages, PPFE may be indistinguishable from a pulmonary apical cap. The latter represents a localized area of subpleural fibrosis, more commonly observed in older individuals and those with a history of smoking, and is typically confined to the uppermost 5 mm of each hemithorax. In contrast, PPFE involves the subpleural regions of the upper lobes, is often asymmetric, and may extend caudally over time. The absence of sclerotic pleural plaques with associated alveolar collapse favors the diagnosis of PPFE [23].

Although initial histological and radiological appearances may overlap, longitudinal imaging is pivotal in distinguishing PPFE from an apical cap. Pulmonary apical caps are typically non-progressive, whereas PPFE is characterized by progressive upper-lobe volume loss, increasing density of subpleural opacities, and extension into adjacent lung regions, occasionally involving lower lung zones. Associated volume loss may result in diaphragmatic elevation [24,25]. The distinction between PPFE, pulmonary apical cap, and UIP pattern in CTD-ILD is summarized in Table 1.

In selected cases, a definitive diagnosis of PPFE may be supported by transbronchial lung cryobiopsy. Compared with surgical lung biopsy, cryobiopsy is less invasive; however, data specific to PPFE remain limited, and procedural risks, including pneumothorax, must be carefully weighed against potential diagnostic benefit [23,26].

## 5. PPFE Across Etiologies: What Makes CTD-PPFE Unique?

Clinically, idiopathic PPFE often presents with progressive exertional dyspnea, a non-productive cough and a distinct clinical phenotype in patients with slender habitus and flattened thoracic cage. The clinical course varies with some patients demonstrating a slow progression while others experience rapid functional decline attributable to extensive pulmonary involvement. Irreversible fibroelastic remodeling results in pronounced parenchymal rigidity of the upper lobes, predisposing patients to further mechanical complications [27]. PPFE associated with CTD may differ biologically from the idiopathic form. Proposed pathogenic mechanisms contributing to fibroelastotic remodeling in this setting include immune-mediated lung injury, microangiopathy, and chronic vasculopathy, although direct evidence linking these processes specifically to PPFE remains limited [18,23]. Disease flares and systemic immune activation may influence the trajectory of pulmonary involvement, potentially contributing to heterogeneity in progression. Whether these factors lead to earlier onset of clinically significant PPFE, accelerated complications, or altered therapeutic responsiveness remains uncertain.

PPFE in the context of CTD is often underrecognized, as it may coexist with other CTD-associated ILD patterns, most commonly NSIP and UIP, which are less commonly reported in idiopathic PPFE. Accurate evaluation and identification of CTD-PPFE is essential for both clinicians and patients. More tailored management strategies may be facilitated, with potential influence on clinical course and prognosis [28]. Compared with idiopathic PPFE, the CTD-associated form frequently manifests earlier, highlighting the importance of heightened clinical awareness in at-risk populations. Timely recognition may support risk stratification and closer clinical monitoring, although evidence for improved outcomes with specific therapeutic interventions remains limited [28,29]. Targeted monitoring and individualized management strategies may help refine care in patients with CTD-associated PPFE [29].

## 6. PPFE in CTD-ILD: Epidemiology and Phenotypes

### 6.1. Systemic Sclerosis (SSc)

Systemic sclerosis is a rare, immune-mediated disease characterized by autoantibody production and injury of the small vessels, leading to progressive fibrosis of the skin and visceral organs. Pulmonary involvement, specifically ILD and pulmonary hypertension, constitutes the leading cause of mortality in these patients. In most cases, ILD manifests with an NSIP pattern and less often with UIP [30]. Two cohorts from the United Kingdom and Italy examined the association between SSc, diagnosed by the established classification criteria, and PPFE [31]. A total of 359 patients were included across two rheumatological centers. PPFE was detected in 65 patients, of whom 41 had extensive PPFE. This pattern of lung involvement was linked to longer disease duration and a more severe impairment of lung function. Increased mortality was observed in the combined cohort of patients with PPFE compared with those without PPFE (44% vs. 32%). A strong association between PPFE and independent bronchial abnormalities (33.7 vs. 1.1% *p* < 0.0001) was identified in the cohort, suggesting a non-random association between PPFE and markers of recurrent airway exposure, including infections and inhaled antigens [31]. In a single-center retrospective study, HRCT was used in 105 patients with SSc-associated ILD to evaluate the presence of PPFE. The prevalence was 18.1% with thirteen patients classified as having “definite” PPFE and six as “consistent with” PPFE. Patients with PPFE were older and had a longer disease duration. In terms of lung function, median FVC was lower in patients with PPFE. Enomoto et al. further proposed the presence of PPFE as an independent predictor of respiratory-related mortality [5]. These findings should be interpreted in the context of predominantly retrospective study designs and relatively small sample sizes, which may limit the robustness of observed associations. Another retrospective study including 11 patients with SSc and PPFE reported radiological findings consistent with a pattern of UIP in most patients and NSIP in two patients, accompanied by an increased rate of emphysema presence. Pulmonary function declined over a two-year follow-up period, indicating a poorer prognosis [7]. Interpretation of these associations is limited by the heterogeneity of definitions, as distinctions between histologically confirmed PPFE, radiological PPFE, and PPFE-like lesions are not consistently applied across studies.

### 6.2. Rheumatoid Arthritis (RA)

The main challenge in patients with RA-ILD is differentiating true PPFE from UIP with atypical or extended distribution involving the upper lobes. Although UIP is typically basal and peripheral in radiological distribution, more extensive or atypical disease may involve the upper lobes and produce pleural abnormalities that can mimic PPFE on CT. In contrast, in RA, this pattern typically involves pleural and subpleural fibroelastosis predominantly affecting the upper lobes, with limited lower-lobe involvement [9]. Cases of PPFE in patients with RA have also been reported. A systematic literature review identified PPFE as a rare pulmonary manifestation, approximately 12% of reported cases of rheumatic autoimmune disease-associated PPFE [7]. Exertional dyspnea and reduced lung volumes are typically present in these patients, while radiological and histopathological findings of upper-lobe-predominant pleural and subpleural fibroelastosis support the diagnosis [28]. Another study including two cohorts of 477 patients with RA-ILD reported a PPFE prevalence of 6.5%. This pattern was associated with a higher rate of complications, particularly pneumothorax, and with greater decline in lung function. Pneumothorax occurred significantly more frequently in patients with PPFE than in those without (28.6% vs. 6.1%; *p* = 0.012) and recurrences were also more common. The increased susceptibility to pneumothorax may relate to characteristic pleural fragility and apical volume loss, which are more often observed in PPFE but are less prominent in classic UIP [8]. Importantly, heterogeneity in imaging criteria and cohort composition across studies makes direct comparison challenging and may contribute to variability in reported prevalence. Reported prevalence estimates should be interpreted cautiously, given that several studies do not consistently differentiate between histologically confirmed PPFE, radiological PPFE, and PPFE-like lesions.

### 6.3. Other Connective Tissue Diseases and Rare Associations

Associations between PPFE and other connective tissue diseases, including primary Sjögren’s syndrome, mixed connective tissue disease, systemic lupus erythematosus, and psoriasis, have been reported, but evidence is limited to small case series or isolated reports. In these settings, PPFE frequently coexists with other ILD patterns and may be radiologically indistinguishable, complicating definitive classification. Most descriptions rely on HRCT findings rather than histopathologic confirmation, further limiting interpretability. Consequently, robust conclusions regarding prevalence, natural history, or prognostic impact cannot be drawn [28,32,33].

Spondyloarthropathies, particularly ankylosing spondylitis, are associated with extra-articular manifestations, with pulmonary involvement being a recognized feature. Patients may develop pleuropulmonary abnormalities, most commonly apical fibrosis, pleural thickening, and upper-lobe fibrobullous disease. These changes typically show upper-lobe predominance, bilateral symmetry, and a progressive course, features that may resemble those observed in PPFE, although they likely reflect distinct underlying processes [34,35].

In conclusion, available studies suggest that PPFE is a rare but clinically important phenotype within CTD-ILD. Excessive elastic fiber deposition in connective tissue diseases may reflect shared immune-mediated and vascular injury pathways, although causality remains unproven. In systemic sclerosis, PPFE has been associated with poorer outcomes and lung volume loss, while in rheumatoid arthritis the risk of pneumothorax appears markedly increased [36]. A structured comparison between idiopathic PPFE and CTD-associated PPFE is presented in Table 2.

## 7. Pathogenesis: A Hypothesis of Vascular and Lymphatic Contribution

The biological mechanisms underlying PPFE remain incompletely understood, and most proposed pathways are extrapolated from other fibrosing interstitial lung diseases rather than being disease-specific. Therefore, to better understand the mechanisms underlying PPFE in the context of connective tissue disease, investigation of circulating biomarkers and their associated pathogenetic pathways is informative. Pleural and subpleural fibroelastic remodeling may arise from repetitive epithelial injury through multiple converging mechanisms. However, most mechanistic insights are extrapolated from broader CTD-ILD and systemic sclerosis studies rather than from PPFE-specific investigations. In the setting of immune dysregulation, these processes may favor aberrant fibroblast activation and impaired tissue repair.

Krebs von den Lungen-6 (KL-6) is a mucin-like glycoprotein primarily expressed by injured regenerating type II alveolar epithelial cells and bronchiolar epithelial cells and is widely used as a circulating biomarker of epithelial injury and disease activity in interstitial lung diseases. In fibrosing ILDs, including CTD-ILD, serum KL-6 levels are typically elevated and correlate with disease extent and progression. Beyond its role as a biomarker, KL-6 has also been implicated in fibrogenic pathways, including myofibroblast activation, although this is primarily based on data from other fibrosing interstitial lung diseases and its relevance to PPFE remains uncertain [37]. However, in idiopathic PPFE, KL-6 levels are often within the normal range or only mildly elevated [24,38]. This relative dissociation suggests that KL-6 may not fully reflect the dominant pathological processes in PPFE. Notably, KL-6 levels tend to increase in patients with concomitant lower-lobe fibrosis, particularly UIP, indicating that biomarker elevation may reflect coexisting fibrosing ILD rather than PPFE itself [38,39]. Despite this limitation, elevated KL-6 has been associated with worse survival and incorporated into prognostic models, with proposed thresholds identifying higher-risk patients [39,40,41]. In CTD-associated disease, longitudinal increases in KL-6 have also been linked to functional decline, supporting its role as a dynamic marker of disease progression [13].

Similarly, surfactant protein D (SP-D), another biomarker produced by type II pneumocytes and typically elevated in CTD-ILD, has been associated with epithelial stress and injury [42,43]. However, as with KL-6, its role in PPFE remains uncertain, and available data do not consistently demonstrate the same degree of elevation observed in other fibrosing interstitial lung diseases. In addition to epithelial-derived proteins, increased circulating levels of endothelial activation markers have been reported in CTD-ILD, particularly in rheumatoid arthritis. Molecules such as E-selectin, intercellular adhesion molecule 1 (ICAM-1), and endothelin-1 are released by activated endothelial cells in response to pro-inflammatory cytokines and promote leukocyte adhesion and transmigration. This endothelial activation may contribute to interstitial inflammation and fibrotic remodeling, although its role in elastotic changes remains unclear; however, its direct contribution to PPFE remains inferential [43].

Monocyte recruitment may further contribute to sustained epithelial stress. In CTDs, increased circulating monocytes and myeloid dendritic cells are associated with heightened toll-like receptor (TLR) expression. In systemic sclerosis, TLR4-mediated signaling enhances transforming growth factor-β responses, a central pathway in fibrogenesis. This may impair effective epithelial repair and favor persistent fibroelastic remodeling, although specific mechanistic data in CTD-PPFE are lacking [44]. Accordingly, the pathways discussed below should be interpreted primarily as biologically plausible mechanisms rather than PPFE-specific processes established directly in CTD-PPFE populations.

Alternatively activated macrophages (M2 phenotype) have also been implicated in CTD-ILD. These cells produce pro-fibrotic mediators, including platelet-derived growth factor, transforming growth factor-β, and the chemokine CCL18. Chronic inflammatory activation in CTDs may promote accumulation of M2 macrophages within the lung, increasing circulating and local levels of these mediators and contributing to excessive extracellular matrix deposition [45].

Precursors of M2 macrophages, including CD16^+^ monocytes, are increased in several CTDs, particularly systemic sclerosis, providing a potential link between monocyte-macrophage polarization and fibroelastotic remodeling in PPFE, though this association has not been directly demonstrated in PPFE cohorts [46]. In parallel, macrophages, activated T cells, and fibroblasts produce interleukin-6 (IL-6), which is elevated in CTD patients. IL-6 promotes Th2 polarization, suppresses Th1 responses, and enhances transforming growth factor-β (TGF-β) signaling. Additional cytokines, such as tumor necrosis factor-α and interleukin-8, increase oxidative epithelial stress and alveolar-capillary permeability, facilitating inflammatory cell recruitment and dysregulated tissue repair, mechanisms that may plausibly contribute to fibroelastotic remodeling but remain unproven in CTD-PPFE [45,47].

**Figure 1:**
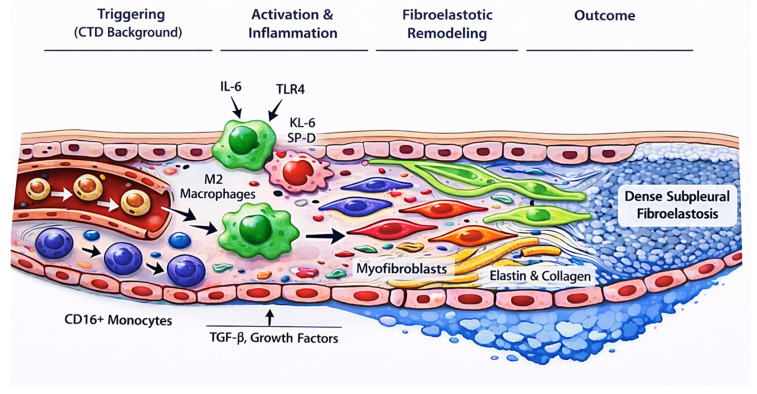
Proposed mechanistic cascade linking connective tissue disease-associated immune activation to pleuroparenchymal fibroelastosis (PPFE). CTD-related immune and vascular dysregulation may drive epithelial injury and fibroblast activation, promoting elastin and collagen deposition within the subpleural lung and resulting in fibroelastosis. The schematic reflects mechanistic plausibility and not established causation.

Finally, the role of vascular endothelial growth factor (VEGF) in PPFE remains incompletely defined. VEGF exerts both pro- and antifibrotic effects, and its dysregulation may contribute to pulmonary fibrosis through paradoxical mechanisms. In CTDs, elevated VEGF levels have been associated with increased disease activity, yet chronic inflammation may result in impaired functional angiogenesis despite high circulating levels. This imbalance between angiogenic signaling and effective vascular repair may favor fibrotic remodeling and warrants further investigation [42,43]. Moreover, lymphatic dysfunction and aberrant lymphangiogenesis have been implicated in fibrotic lung diseases and may be particularly relevant in PPFE given its subpleural predilection, yet direct mechanistic validation in CTD-PPFE populations has not been established [42,43]. The principal candidate pathways potentially linking connective tissue disease-associated ILD to fibroelastotic remodeling in PPFE are summarized in Table 3 and the proposed biological cascade linking connective tissue disease-associated immune activation to subpleural fibroelastotic remodeling is schematically illustrated in Figure 1.

Beyond molecular drivers, the consistent spatial distribution of PPFE raises the question of regional susceptibility. Histologic and imaging series consistently demonstrate early subpleural apical involvement with progressive upper-lobe volume loss [2,23]. Several non-mutually exclusive mechanisms may contribute. Apical lung regions are exposed to higher transpulmonary pressures, lower perfusion, and greater mechanical stress gradients, potentially increasing vulnerability to repetitive microinjury and aberrant repair [1,2]. In CTD-ILD, chronic immune-mediated epithelial and microvascular injury may amplify this regional susceptibility [5,6]. Furthermore, reports of telomere-related dysfunction in PPFE cohorts suggest impaired reparative capacity in mechanically stressed subpleural regions [23]. Taken together, these observations are best viewed as hypothesis-generating and help explain biological plausibility, but they do not yet define a mechanism specific to CTD-associated PPFE. Overall, these observations suggest that multiple pathways may be involved in PPFE pathogenesis; however, their relative contribution and spatial distribution within the lung remain unclear.

## 8. Prognostic Implications

Early recognition of PPFE is important to limit its adverse clinical impact. PPFE has been associated with impaired lung function, most notably a rapid decline in forced vital capacity, often with relative preservation of residual volume. Two patterns of FVC decline have been described: a rapid course over 2-6 years and a more indolent trajectory evolving over longer periods [23]. Total lung capacity is typically reduced, while the ratio of forced expiratory volume in 1 s to FVC may be increased. This restrictive physiology is commonly accompanied by a reduction in diffusing capacity for carbon monoxide [36].

Assessment of disease progression in PPFE also presents challenges. Although FVC decline is commonly used to define progression in fibrosing ILD, it may underestimate disease severity in PPFE. This reflects the unique physiological profile of the disease, in which predominant upper-lobe fibrosis, progressive pleural thickening, and chest wall restriction lead to disproportionate reductions in total lung capacity with relative preservation or even increase in residual volume. As a result, FVC may remain relatively stable despite ongoing structural progression. In this context, parameters such as residual volume (RV) and the residual volume/total lung capacity (RV/TLC) ratio may better capture disease-related physiological impairment, although their role in longitudinal monitoring has not been systematically established [24].

Progressive upper-lobe fibrosis with extension into adjacent lung regions results in ongoing lung volume loss and diaphragmatic elevation, contributing to worsening functional impairment [33]. Recognition of PPFE also has implications for complication prevention and surveillance. Pneumothorax, pneumomediastinum, and opportunistic infections occur more frequently in patients with PPFE, reflecting parenchymal fragility and associated chest wall and diaphragmatic abnormalities [48]. Notably, the prognostic trajectory of PPFE is shaped not only by parenchymal fibrosis but also by its distinctive mechanical features, including upper-lobe volume loss, chest wall restriction, and a high propensity for pneumothorax, which together contribute to disproportionate functional decline and clinical deterioration, while recurrent infections further compound this risk. Chronic pulmonary infections with opportunistic pathogens, particularly Aspergillus species, nontuberculous mycobacteria, and Pseudomonas aeruginosa, are also more prevalent in this population [49,50,51].

Overall outcomes in PPFE are generally unfavorable, as the combined burden of progressive functional decline and complications is associated with poorer prognosis [31]. However, the strength of this evidence remains limited, as most available data arise from observational studies with heterogeneous definitions, and prospective, standardized studies are lacking. In addition, many studies do not clearly distinguish between histologically confirmed PPFE, radiological PPFE, and PPFE-like lesions. Higher rates of respiratory-related mortality have been reported, particularly in CTD-associated PPFE, where progressive parenchymal fibrosis and declining lung function increase the risk of respiratory failure [11,31]. In addition, recurrent infections in patients with already compromised lung parenchyma may further worsen prognosis and contribute to mortality [31].

From a clinical perspective, PPFE should be suspected in CTD-ILD patients with disproportionate upper-lobe pleural and subpleural fibrosis on HRCT, particularly when accompanied by progressive volume loss, recurrent pneumothorax, or functional decline that appears discordant with the extent of lower-lobe disease. Recognition of this pattern may warrant closer functional monitoring, surveillance for complications, and multidisciplinary evaluation, although evidence to guide specific management strategies remains limited.

## 9. Future Directions

Despite increasing recognition, several critical gaps remain in the understanding and management of PPFE in CTD-ILD. A major limitation across existing studies is the lack of standardized radiological and clinical criteria for defining PPFE and PPFE-like lesions, resulting in heterogeneous prevalence estimates and inconsistent prognostic associations. Future studies should prioritize the development and validation of uniform HRCT-based scoring systems, ideally incorporating longitudinal imaging to distinguish progressive PPFE from static apical abnormalities, alongside standardized definitions that differentiate histologically confirmed PPFE, radiological PPFE, and PPFE-like lesions, and prospective designs to better define prevalence, natural history, and prognostic impact [10,11].

Earlier recognition represents another key unmet need. Diagnostic delay is common, particularly when PPFE evolves slowly or coexists with more typical CTD-ILD patterns, leading to prolonged diagnostic odysseys and missed opportunities for risk stratification. Illustrative cases highlight how delayed recognition of upper-lobe fibroelastotic disease can span many years despite repeated specialist evaluations, underscoring the importance of heightened clinical suspicion and multidisciplinary assessment in patients with unexplained progressive dyspnea and upper-lobe volume loss [39,52].

Prospective cohort studies are also needed to clarify the natural history of CTD-associated PPFE, including its temporal relationship with underlying ILD patterns, rates of functional decline, and drivers of complications such as pneumothorax and infection. In particular, whether infection risk differs between idiopathic and CTD-associated PPFE remains unknown, as existing studies do not provide stratified analyses. This is clinically relevant given the potential impact of immunosuppressive therapies in CTD-ILD populations, although PPFE-specific data are lacking. Integration of circulating biomarkers reflecting epithelial injury, endothelial activation, and immune dysregulation may help identify patients at higher risk of progression and facilitate earlier phenotypic classification [37,42,43]. In addition, there are no data to support routine use of prophylactic antibiotics in PPFE, and such approaches are currently extrapolated from broader CTD-ILD practice [53].

Mechanistically, further investigation into vascular and lymphatic pathways is warranted. The relative contribution of endothelial dysfunction, impaired angiogenesis, and aberrant lymphatic remodeling to fibroelastotic progression remains poorly defined, particularly in CTD-PPFE. Translational studies combining imaging, tissue analysis, and molecular profiling could help determine whether PPFE represents a distinct biological phenotype or a regional manifestation of systemic fibrotic susceptibility.

Finally, therapeutic responsiveness remains largely unexplored, and no disease-specific treatment strategies exist for PPFE. Management is therefore largely extrapolated from broader CTD-ILD practice, with supportive care playing a central role, including optimization of oxygen therapy, pulmonary rehabilitation, and attention to nutritional status, as patients are frequently underweight and may experience progressive weight loss [53]. This has important implications for treatment tolerability, particularly with antifibrotic agents, where gastrointestinal adverse effects may further compromise nutritional status. While antifibrotic and immunomodulatory therapies are increasingly used in fibrosing ILD phenotypes, their role in PPFE remains uncertain and evidence is limited. Carefully designed observational studies and pragmatic trials will be essential to inform evidence-based management strategies. 

## 10. Conclusions

Pleuroparenchymal fibroelastosis represents a distinct and clinically meaningful phenotype within the spectrum of connective tissue disease-associated interstitial lung disease. Although rare, PPFE is increasingly recognized due to improved imaging and greater awareness, particularly in systemic sclerosis and rheumatoid arthritis. Accumulating evidence indicates that its presence is associated with accelerated functional decline, increased complications, and poorer respiratory outcomes.

Current data suggest that PPFE in CTD-ILD arises from a convergence of repetitive epithelial injury, immune-mediated inflammation, and vascular dysfunction, with emerging evidence implicating aberrant lymphatic remodeling. However, causality remains unproven, and mechanistic pathways are incompletely understood. Current evidence is also constrained by heterogeneity in diagnostic definitions and predominantly observational study designs, and caution is required when interpreting reported prevalence and prognostic associations. Frequent overlap with other ILD patterns and delayed recognition continue to limit timely phenotypic classification.

Improved standardization of diagnostic criteria, earlier identification through longitudinal imaging and multidisciplinary evaluation, and focused investigation of endothelial and lymphatic pathways are essential next steps. Advancing understanding of CTD-associated PPFE will be critical to refining prognostication, guiding surveillance for complications, and ultimately developing targeted therapeutic approaches for this vulnerable patient population.

## Figures and Tables

**Table 1:** Distinguishing pleuroparenchymal fibroelastosis (PPFE) from pulmonary apical cap and usual interstitial pneumonia (UIP) pattern in connective tissue disease-interstitial lung disease (CTD-ILD). Comparison of key clinical, clinicopathological and radiological features. PPFE is characterized by upper-lobe-predominant pleural and subpleural fibroelastosis with potential progression and complications, whereas apical cap represents a localized, non-progressive subpleural scar, and UIP typically shows basal-predominant fibrosis with a progressive course.

Feature	PPFE	Pulmonary Apical Cap	UIP Pattern in CTD-ILD
**Primary Location**	Visceral pleura and subpleural upper lobes	Apex (<5 mm from pleura)	Basal and subpleural predominance
**Histological Pattern**	Elastin and collagen	Scar tissue	Fibrosis with architectural distortion and honeycombing
**Radiological Pattern**	Dense pleural thickening with subpleural fibrosis (upper lobes)	Thin apical fibrotic cap	Reticular pattern with honeycombing (basal predominance)
**Progression**	Progressive downward extension	Non-progressive	Progressive, without directional extension
**Complications**	Pneumothorax, infections	Rare	Acute exacerbations, pulmonary hypertension

**Table 2:** Comparative features of idiopathic PPFE (IPPFE) and connective tissue disease-associated PPFE (CTD-PPFE). Comparison between idiopathic PPFE and CTD-associated PPFE in terms of etiology, proposed pathobiology, associated interstitial lung disease patterns, and clinical outcomes. CTD-PPFE is more frequently associated with coexisting fibrotic ILD patterns and appears to carry a less favorable prognosis.

Feature	IPPFE	CTD-PPFE
**Etiology**	Rare idiopathic interstitial pneumonia	Existing in patients with CTD (systemic sclerosis, rheumatoid arthritis, Sjögren’s syndrome)
**Mechanism**	Not identified	Immune-mediated lung injury, microangiopathy and chronic vasculopathy
**Coexisting ILD patterns**	Rarely	Often coexisting NSIP and UIP
**Prognosis**	Slow progression but varies	Poorer outcomes, reduced survival and increased rates of complications

**Table 3:** Proposed mechanistic pathways contributing to PPFE in connective tissue disease-associated ILD. Summary of candidate biological pathways potentially linking connective tissue disease-associated ILD (CTD-ILD) to fibroelastotic remodeling. These mechanisms are extrapolated from CTD-ILD and systemic sclerosis pathobiology and provide mechanistic plausibility rather than direct causal evidence in PPFE.

Mechanism	Involved Biomarker/Cell	Observed Action/Correlation in CTD-ILD	Potential Role in PPFE
**Epithelial** **Injury** [37,39,42]	KL-6, SP-D	Elevated levels; reflect type II pneumocyte damage	Promoting myofibroblast differentiation, initiating fibroelastotic process
**Endothelial** **Activation** [43]	E-selectin, ICAM-1, Endothelin-1	Released by activated endothelial cells, leukocyte adhesion	Facilitating inflammation, vascular remodeling, tissue ischemia
**Immune Dysregulation** [45]	M2 Macrophages, IL-6, TLR4	Increased pro-inflammatory and pro-fibrotic mediators	Enhancing TGF-β signaling, perpetuating abnormal wound healing
**Vascular** **Dysfunction** [42,43]	VEGF	Paradoxical regulation, insufficient angiogenesis	Tissue hypoxia, impaired fluid clearance, subpleural space remodeling

## Data Availability

No new data were generated or analyzed in this study. All information discussed in this article is derived from previously published literature cited in the reference list.

## Abbreviations

The following abbreviations are used in this manuscript:

ANCA	Antineutrophil Cytoplasmic Antibodies
CTD	Connective Tissue Disease
CTD-ILD	Connective Tissue Disease-associated Interstitial Lung Disease
FVC	Forced Vital Capacity
HRCT	High-Resolution Computed Tomography
ICAM-1	Intercellular Adhesion Molecule 1
IL-6	Interleukin-6
ILD	Interstitial Lung Disease
KL-6	Krebs von den Lungen-6
NSIP	Nonspecific Interstitial Pneumonia
PPFE	Pleuroparenchymal Fibroelastosis
RA	Rheumatoid Arthritis
RV	Residual Volume
SANRA	Scale for the Assessment of Narrative Review Articles
SP-D	Surfactant Protein D
SSc	Systemic Sclerosis
TGF-β	Transforming Growth Factor-β
TLR	Toll-like Receptor
TLC	Total Lung Capacity
UIP	Usual Interstitial Pneumonia
VEGF	Vascular Endothelial Growth Factor
